# Psychometric Evaluation of Chinese-Language 44-Item and 10-Item Big Five Personality Inventories, Including Correlations with Chronotype, Mindfulness and Mind Wandering

**DOI:** 10.1371/journal.pone.0149963

**Published:** 2016-02-26

**Authors:** Richard Carciofo, Jiaoyan Yang, Nan Song, Feng Du, Kan Zhang

**Affiliations:** 1 Key Laboratory of Behavioral Science, Institute of Psychology, Chinese Academy of Sciences, Beijing, China; 2 Language Centre, Xi’an Jiaotong-Liverpool University, Suzhou, China; 3 School of English for Specific Purposes, Beijing Foreign Studies University, Beijing, China; Southwest University, CHINA

## Abstract

The 44-item and 10-item Big Five Inventory (BFI) personality scales are widely used, but there is a lack of psychometric data for Chinese versions. Eight surveys (total *N* = 2,496, aged 18–82), assessed a Chinese-language BFI-44 and/or an independently translated Chinese-language BFI-10. Most BFI-44 items loaded strongly or predominantly on the expected dimension, and values of Cronbach's alpha ranged .698-.807. Test-retest coefficients ranged .694-.770 (BFI-44), and .515-.873 (BFI-10). The BFI-44 and BFI-10 showed good convergent and discriminant correlations, and expected associations with gender (females higher for agreeableness and neuroticism), and age (older age associated with more conscientiousness and agreeableness, and also less neuroticism and openness). Additionally, predicted correlations were found with chronotype (morningness positive with conscientiousness), mindfulness (negative with neuroticism, positive with conscientiousness), and mind wandering/daydreaming frequency (negative with conscientiousness, positive with neuroticism). Exploratory analysis found that the Self-discipline facet of conscientiousness positively correlated with morningness and mindfulness, and negatively correlated with mind wandering/daydreaming frequency. Furthermore, Self-discipline was found to be a mediator in the relationships between chronotype and mindfulness, and chronotype and mind wandering/daydreaming frequency. Overall, the results support the utility of the BFI-44 and BFI-10 for Chinese-language big five personality research.

## Introduction

Over the last few decades many questionnaire-based and lexical studies have led to international personality research being dominated by the Big Five model of personality, involving broad domains/dimensions of extraversion, agreeableness, conscientiousness, neuroticism (emotional stability), and openness to experience (intellect), with each having several facets in a hierarchical structure [1, 2, 3, 4, 5]. Questionnaire assessment of the big five (for discussions, see [2, 3]), includes the revised, 240-item NEO Personality Inventory (NEO-PI-R), which assesses six facets for each dimension, and the shortened NEO Five Factor Inventory (NEO-FFI) with 12 items per dimension (see [2, 6]). However, the NEO-PI-R and the NEO-FFI are proprietary instruments which limits their availability for research [7, 8]. Alternatives, freely available (for non-commercial use), include the International Personality Item Pool (IPIP [7]) big five factor markers, and the 44-item Big Five Inventory (BFI-44 [2, 3, 9]). The BFI-44 was developed as a quick assessment of the core aspects of each big five domain, and has shown a clear five-factor structure, reliability, convergent validity with other big five scales (including the NEO-PI-R and NEO-FFI), and strong self-peer agreement [2, 3, 5, 10]. Its five-factor structure has been substantially replicated in all major world regions [11]. Also, from the BFI-44 items two facets for each big five domain can be assessed (for example, extraversion includes the facets of 'assertiveness' and 'activity'), and these have been shown to have strong convergence with the corresponding facets assessed with the NEO-PI-R [5].

The five-factor model has been replicated in Asian countries, including China [2]. For example, McCrae et al. [12] and Yang et al. [13] replicated the five-factor structure of the NEO-PI-R with Chinese samples, and Zheng et al. [14] replicated the five-factor model with Chinese-language IPIP big five factor markers. However, although Chinese versions of the BFI-44 have also been used in research, detailed psychometric information has typically not been reported. For example, Zheng et al. [14] refer to use of the BFI-44 with Chinese participants in their validation of Chinese IPIP big five factor markers, but do not identify the provenance of the translation or detail its psychometric properties. Although Leung et al. [15] developed a new translation of the BFI-44 in research with Hong Kong-based participants, their translation used traditional Chinese characters which limits its usefulness in mainland China where simplified characters are more widely used. The first aim of the current research was thus to provide psychometric evaluation of a specified Chinese translation of the BFI-44 utilising simplified Chinese characters.

While scales such as the NEO-PI-R and BFI-44 continue to be widely used, there has also been a recent trend towards the development of much shorter scales, which, while inevitably having poorer psychometric properties in certain respects, involve a balance between practical and psychometric considerations, and can save time and help avoid the frustration and boredom associated with completing longer scales [16, 17, 18, 19, 20]. Gosling et al. [16] developed a Ten-Item Personality Inventory (TIPI), with two items for each big five dimension, which showed good test-retest reliability, convergent validity with the BFI-44, criterion-related validity, and consistency with peer-ratings. Many translations have been made, including German [19, 21], Dutch [22], Spanish [23], and Japanese (see [24]). Another 10-item scale (two per dimension) is the BFI-10, developed by Rammstedt and John [10] in English and German versions, utilising items taken directly from the BFI-44. The BFI-10 scales have shown good test-retest reliability, convergent validity (with the NEO-PI-R), external validity (with peer ratings), and a five-factor structure [10]. In addition, Thalmayer et al. [8] found that the BFI-10 has comparable (or even superior) predictive validity to the BFI-44 for various measures, including grade point average (GPA) which was significantly predicted by conscientiousness. They also compared the original BFI-10 with alternate versions comprised of other randomly selected BFI-44 items, and found that the original showed best predictivity, indicating that the best/most valid items had been selected in developing the BFI-10. The second aim of the current research was thus to provide psychometric evaluation of a Chinese version of the BFI-10.

The current psychometric evaluation included assessment of the construct validity of the BFI-44 and BFI-10 scales, through a nomological network of predicted external correlates [16]. The establishment of cross-cultural comparability of personality measures can also be facilitated when "… functional equivalence can be demonstrated by showing the trait scales relate to external variables in similar ways." [11, p.175]. The current research assessed associations with age, gender, chronotype (morningness-eveningness preference), mindfulness (“…the state of being attentive to and aware of what is taking place in the present” [25, p.822]), and mind wandering/daydreaming (distraction of attention away from the external environment, and any ongoing task, towards internally focused mentation [26, 27]).

There have been some mixed findings regarding age and gender differences in big five personality dimensions, which may be partly related to factors such as study design, measures (questionnaires) used, cohort effects, etc [28]. However, for age differences, previous longitudinal and cross-sectional research has typically found that (between early adulthood and middle age) agreeableness and conscientiousness are positively associated with increasing age, while neuroticism and openness are negatively associated, and extraversion is relatively stable [6, 13, 28, 29, 30]. For gender differences, the most consistently reported findings have been that females score higher for agreeableness and neuroticism [28]. For example, with samples from 55 nations (*N* = 17,637), Schmitt et al. [31] found that females scored significantly higher than males: in 49/55 nations for neuroticism (men did not score significantly higher in any); in 34/55 nations for agreeableness (males significantly higher in 1/55); in 25/55 nations for extraversion (males significantly higher in 2/55); in 23/55 nations for conscientiousness (males significantly higher in 2/55); and in 4/55 nations for openness (males significantly higher in 8/55).

Chronotype (morningness-eveningness preference), was also included as an external correlate in the nomological network, as previous research indicates that a correlation with conscientiousness can be expected. Tsaousis' [32] meta-analysis of 31 studies found that conscientiousness is the strongest personality correlate of chronotype (positive with morningness; *r* = .29); agreeableness ranked second (*r* = .13), with weak correlations for openness (-.09), neuroticism (-.07), and extraversion (-.06). Additionally, Hogben et al. [33] found that conscientiousness is the strongest predictor of chronotype after controlling for variables including age, gender and sleep disorders. Previous research also indicates the correlations that can be expected between personality and mindfulness. Giluk's [34] meta-analysis found that mindfulness has a strong negative correlation with neuroticism (*r* = -.45), moderate positive correlations with conscientiousness (.32) and agreeableness (.22), and weaker positive correlations with openness (.15) and extraversion (.12).

Although there has been little research on personality correlates of mind wandering or daydreaming since the development of the big five personality model, these variables were also included in the nomological network, because some predictions are suggested by the patterns of inter-correlation with chronotype and mindfulness. Mind wandering/daydreaming frequency negatively correlates with mindfulness [35, 36, 37], while morningness positively correlates with mindfulness [38], but negatively correlates with mind wandering/daydreaming frequency [37, 39]. Consistently, Jackson and Balota [40] found that mind wandering frequency is negatively correlated with conscientiousness (while, as noted above, conscientiousness positively correlates with morningness). Also, morningness is associated with positive affect [41], as is mindfulness [34, 38], while mind wandering/daydreaming frequency are associated with negative affect [39, 42, 43, 44], suggesting a possible positive correlation with neuroticism.

Finally, as the BFI-44 questionnaire allows for assessment of two facets for each big five dimension [5], a third aim of the current research was to undertake an exploratory study of how these facets are associated with chronotype, mindfulness, mind wandering, and daydreaming frequency.

## Method

### Materials

The Chinese version of the BFI-44 used in the current research was that reproduced in the Chinese translation of John and Srivastava [3]. Each item is assessed on a 5-point Likert scale with anchors of 'disagree strongly' (1) to 'agree strongly' (5); some items are reverse-scored (see Results section for details). Extraversion has 8 items, giving a range of 8–40; agreeableness has 9 items (range 9–45); conscientiousness has 9 items (range 9–45); neuroticism has 8 items (range 8–40); and openness has 10 items (range 10–50).

The BFI-10 consists of 10 items taken from the BFI-44, with two for each big five domain (one reverse-scored): extraversion (items 6, 36); agreeableness (items 2, 22); conscientiousness (items 3, 23); neuroticism (items 9, 39); openness (items 20, 41). Each item is assessed with the same Likert scale as for the BFI-44, giving a range of 2–10 for each domain total score. A Chinese translation of the BFI-10 (see S1 Appendix) was made directly from the English BFI-10 presented in Rammstedt and John [10]. The translation was made by native Chinese-speakers, then back-translated by another native Chinese-speaker, and then checked against the original English by a native English-speaker. Errors were corrected and the translations changed accordingly. This BFI-10 translation had been made prior to obtaining the BFI-44 reproduced in the Chinese edition of John and Srivastava [3]. Comparison of the items in this independent Chinese BFI-10 with the corresponding items in the Chinese BFI-44 from John and Srivastava [3] showed different wording in each case. Thus, the independent BFI-10 could be compared with: a) the full BFI-44; and, b) an alternative BFI-10 extracted from the BFI-44 (the BFI-10X).

External, criterion validity for the BFI-44 and BFI-10 was assessed with Chinese versions of the following scales:-

1. The reduced Morningness-Eveningness Questionnaire (rMEQ [45, 46]), a 5-item version of the 19-item Morningness-Eveningness Questionnaire [47, 48]. Scores range 4–25, with higher scores indicating more morningness.
2. The Daydreaming Frequency scale (DF; 12 items, ranging 12–60; higher = more daydreaming), and the Mind Wandering scale (MW; 12 items, 6 reverse-scored, ranging 12–60; higher = more mind wandering), from the Imaginal Processes Inventory [37, 49].
3. The Mindful Attention Awareness Scale-Lapses Only (MLO [37, 50]), measure of mindfulness (12 items, ranging 12–72; higher = more frequent mindful states). This is a shortened version of the Mindful Attention Awareness Scale [25].
4. The Attention-related Cognitive Errors Scale (CES [37, 50, 51]), measure of action slips/errors (12 items, ranging 12–60; higher = more slips/errors).

For each BFI-44 domain scale, and also for the DF, MW, MLO, and CES scales, a single missing item was replaced by the mean of the other responses for that participant. Questionnaires were excluded if there were two or more omissions, or an error (e.g., multiple answers for an item). For the rMEQ, those with an error or one/more omissions, were excluded. Any individual BFI-10 domain scale/s with error/s, or missing value/s, were excluded.

### Participants and Procedure

The BFI-44 and BFI-10 were administered to consenting participants in independent survey samples (total *N* = 2,496). These surveys were undertaken between May 2012 and November 2013, each involving a different combination of questionnaires, plus demographic information (some surveys also included other scales, not reported here). The sequence of the scales used in each survey was varied, so that the order of presentation was counter-balanced to some degree. For example, for a survey involving the Daydreaming Frequency (DF) scale, 44-item Big Five Inventory (BFI-44), and the reduced Morningness-Eveningness Questionnaire (rMEQ), some participants completed the scales in the order DF/BFI-44/rMEQ, while others completed them in the order rMEQ/BFI-44/DF.

Sample details are shown in Table 1. Participants were mostly full-time or part-time students (of various subjects) from several Beijing universities/institutes, who completed the surveys during class breaks. A few community residents were also sampled. Participation was voluntary and unpaid. Participants either completed the questionnaires anonymously, or gave written informed consent. Ethical approval was obtained from the Internal Review Board of the Institute of Psychology, Chinese Academy of Sciences.

**Table 1:** Sample details.

	*N* (male / female)†	Mean reported age (sd / range)	Mean reported years of education (sd)	Male mean age (sd)	Female mean age (sd)
Study 1a	235 (56 / 179)	25.50 (8.30 / 18–67)	14.18 (2.81)	27.72 (10.63)	24.80 (7.32)
Study 1b	186 (72 / 114)	31.36 (6.41 / 18–48)	15.94 (3.96)	32.50 (6.43)	30.65 (6.32)
Study 1c	172 (68 / 104)	27.75 (5.78 / 18–52)	15.31 (4.45)	29.22 (6.93)	26.76* (4.63)
Study 1d	214 (116 / 93)	24.64 (5.75 / 18–45)	14.34 (3.13)	26.35 (4.86)	22.43*** (6.07)
Study 2a	1019 (319 / 692)	26.95 (9.77 / 18–82)	15.00 (3.72)	30.13 (9.23)	25.49*** (9.67)
Study 2b	213 (74 / 139)	23.96 (5.42 / 18–41)	14.45 (2.97)	27.03 (5.79)	22.33*** (4.43)
Study 2c	189 (52 / 137)	23.78 (6.77 / 18–50)	14.06 (3.56)	25.96 (8.52)	22.96* (5.79)
Study 2d	268 (74 / 194)	18.90 (.789 / 18–21)	13.18 (1.84)	19.14 (.782)	18.81** (.775)

^†^*N*-value discrepancies are due to missing data. Gender difference t-test significant:

*p ≤ .05;

**p ≤ .01;

***p ≤ .0005.

For *Study 1a* the BFI-44, BFI-10 and rMEQ were completed. The BFI-10 was added to the end of the BFI-44, producing a 54-item scale. A sub-group (*n* = 91) completed a retest 5–6 weeks after the first survey. *Study 1b* involved the DF scale, BFI-44, and rMEQ; *Study 1c* involved the MW scale, BFI-44, and rMEQ; *Study 1d* involved the MLO scale, BFI-44, and rMEQ.

For *Study 2a* each participant completed one of six separate surveys, each involving the BFI-10 and rMEQ, plus different combinations of questionnaires in each survey: for DF, *n* = 363; MW, *n* = 198; MLO, *n* = 193; CES, *n* = 265. These were part of a larger series of surveys, with a total of *N* = 1852, reported in Carciofo et al. [37]. *Study 2b* involved the sample reported in Carciofo et al. [39, Study 1]. The BFI-10, DF scale and rMEQ were completed. A sub-group (*n* = 79) provided test-retest data for the BFI-10, approximately 5 weeks after the first survey. *Study 2c* involved a sub-sample of that reported in Carciofo et al. [39, Study 2]. The BFI-10, MW scale and rMEQ were completed. A sub-group (*n* = 91) provided test-retest data for the BFI-10, approximately 5 weeks after the first survey. *Study 2d* involved a sub-sample of that reported in Carciofo et al. [39, Study 3]. The BFI-10, DF scale, MW scale, and rMEQ were completed (the BFI-10 and MW scale were administered seven days after the DF scale and rMEQ). A sub-group (*n* = 174) also completed the MLO scale 4–6 weeks after completing the BFI-10.

### Statistical analysis

There has been discussion about the optimal approach to the analysis of scale structure in personality research (e.g., [12, 28, 52, 53]). Confirmatory factor analysis (CFA) has become widely used (e.g., [15]), but various approaches to exploratory factor analysis (EFA) also continue to be employed (see, e.g., [18, 24]), and it has been shown that EFA and multi-trait, multi-method analysis may support the five-factor model, while CFA (with its more stringent assumptions) may not [28, 52]. Also, although there are issues with the use of principal components analysis (PCA) in conjunction with varimax rotation [54, 55], the big five structure has been replicable using this approach, although large sample sizes may be required [12]. Although neither CFA nor EFA may be appropriate/effective for short scales such as the BFI-10 [18], both approaches have been used as part of psychometric evaluation (e.g., [10, 19, 22]). Following other studies (e.g., [9, 12, 14, 22]) we undertook PCA with varimax rotation for the BFI-44 and the BFI-10. We also note the results of CFA for the BFI-44, to allow for comparisons with other CFA studies, including that of Leung et al. [15].

Descriptive statistics include the mean, standard deviation and Cronbach's coefficient alpha (measure of internal consistency) for each BFI-44 and BFI-10 dimension, in each study. Coefficient alpha is influenced by the number of items in a scale [16, 56], so the BFI-10, with only two items to cover each personality dimension, is likely to have relatively poor values of alpha. Although alpha coefficients may be difficult to interpret for such short scales [17], we include them for comparison with other studies. Test-retest was also undertaken (in studies 1a, 2b, and 2c), which may be more appropriate than coefficient alpha for assessing the reliability of short scales [57]. Correlations were assessed with Pearson product-moment coefficients. Absolute mean average coefficients were calculated using Fisher's *r* to *Z* transformation. Linear regression was used to assess the associations of age and gender with each big five domain. Reported *p*-values are for two-tailed tests.

## Results

### BFI-44 scale structure

PCA with varimax rotation (with Kaiser normalisation) was undertaken on the combined BFI-44 data, excluding cases with any missing data (*N* = 798 for each domain). The first 5 eigenvalues were 7.679, 4.084, 2.906, 2.357, and 2.232; the next two were 1.558, and 1.294. The scree plot (available from the authors) showed a clear break after the 5th component. Only three items loaded > .2 on the 6th component, although these were all for the openness domain and loaded strongly: items 30 (.769), 41 (.809), and 44 (.829). Loadings for the first five components are shown in Table 2. For each dimension most items had strong or predominant loadings on the corresponding component, with five or more of the relevant items loading at least > .3: extraversion (component 5), items 1, 6, 16, 21, 36; agreeableness (component 2), items 7, 17, 22, 27, 32, 42; conscientiousness, all items on component 3; neuroticism (component 4), items 14, 19, 24, 34, 39; openness (component 1), items 5, 10, 15, 20, 25, 40. There were some cross-loadings, such as between extraversion, agreeableness and openness.

**Table 2:** Principal components analysis with varimax rotation for the combined BFI-44 data.

	All BFI-44 studies combined (N = 798)				
	Component				
Item	1	2	3	4	5
e1	**.353**	.160	-.061	-.079	**.585**
e6R	.105	-.015	.031	-.076	**.646**
e11	**.328**	.228	.220	-.060	.202
e16	**.494**	**.361**	.096	.088	**.325**
e21R	-.001	-.006	-.098	-.128	**.715**
e26	**.397**	.050	.193	**-.365**	.073
e31R	.070	-.137	.018	**-.600**	.290
e36	.279	**.315**	-.001	-.174	**.578**
a2R	-.120	.251	.064	-.147	-.148
a7	.221	**.609**	.183	.064	.091
a12R	.004	.285	.155	.091	-.115
a17	**.370**	**.589**	.013	-.064	.039
a22	.088	**.569**	.015	.037	.058
a27R	-.072	**.351**	.120	-.044	**.587**
a32	.093	**.701**	.104	.087	-.016
a37R	.033	.021	.070	-.109	-.003
a42	.052	**.563**	.009	-.069	.212
c3	.121	.198	**.549**	.093	-.045
c8R	-.121	-.132	**.469**	-.260	-.067
c13	.277	**.370**	**.430**	.223	.006
c18R	.015	-.298	**.655**	-.036	.094
c23R	.022	.187	**.688**	-.219	.067
c28	.226	**.364**	**.500**	-.004	-.074
c33	**.345**	.232	**.438**	-.168	-.004
c38	.139	.056	**.471**	-.004	.046
c43R	.031	-.027	**.495**	**-.401**	-.090
n4	-.008	-.106	-.088	.235	**-.399**
n9R	-.294	-.263	-.083	.206	-.065
n14	-.042	.055	-.029	**.685**	-.031
n19	.140	-.172	-.139	**.636**	-.125
n24R	-.008	-.188	-.010	**.315**	.045
n29	.080	-.080	-.142	.295	-.178
n34R	**-.384**	-.185	-.122	**.356**	.084
n39	-.063	.054	-.073	**.620**	-.104
o5	**.819**	.104	.039	-.047	.067
o10	**.492**	**.301**	.047	.232	.144
o15	**.699**	.034	.187	.035	.022
o20	**.768**	.132	-.004	.000	.077
o25	**.766**	.082	.030	-.166	.079
o30	.232	.202	.073	.089	.052
o35R	.097	-.177	-.152	-.192	-.017
o40	**.324**	.155	.111	.196	-.129
o41R	.094	.041	.059	.000	.105
o44	.209	-.022	-.083	-.060	.037

e = extraversion; a = agreeableness; c = conscientiousness; n = neuroticism; o = openness. All absolute loadings > .3 are in **bold**. R = Reverse-scored items (adjusted so that all item loadings for a personality domain should be positive).

The current results show some correspondence with the findings of Leung et al. [15] for their BFI-44 utilising traditional Chinese characters, in the items that had (relatively) poor loadings on the relevant component/factor. In particular agreeableness items 2, 12 and 37, and openness items 30, 35, 41 and 44. Several of these items were reverse-scored (see Table 2). Leung et al. [15] also found that many of their poor-loading items were reverse-scored, and their CFA analysis led them to exclude 15 items, leaving 29 items which showed acceptable/good fit to the five-factor model. The excluded items were (extraversion) e6, e26; (agreeableness) a2, a12, a27, a37; (conscientiousness) c8, c18, c23, c43; (neuroticism) n34; (openness) o30, o35, o41, o44. For comparison, CFA (AMOS v.17, using maximum likelihood estimation, and with co-varied factors) was undertaken on the 29 items retained by Leung et al. [15]. According to Brown [58] and Hooper et al. [59], adequate model fit may be indicated by RMSEA (root mean square error of approximation) < .08; SRMR (standardized root mean square residual) < .08; CFI (Comparative Fit Index) > .90; and relative/normed Chi-square (Chi-squared statistic/degrees of freedom) < 5.0. The CFA showed: relative/normed Chi-square = 5.817, CFI = .767, and SRMR = .0896, indicating poor model fit, although the RMSEA of .078 (90% confidence interval = .075-.081) was (just) within the acceptable range. However, for the full BFI-44, although poor model fit was again indicated by relative/normed Chi-square = 5.979, CFI = .616, and SRMR = .0964, RMSEA was again reasonable, .079 (90% confidence interval = .077-.081). Also, some of the poor-loading items in Leung et al.'s [15] analysis had moderate/strong loadings on the corresponding component in the current study (e.g., items e6, c8, c18, c23). Further testing of the structure of the BFI-44 seems warranted to give a stronger basis for any modification or deletion of items (see Discussion).

### BFI-10 scale structure

PCA with varimax rotation (with Kaiser normalisation) for the complete BFI-10 data (*N* = 1853 for each domain) extracted 5-components (Table 3): e1 (.891) and e6 (.762) loaded on Component 1 (C1); o5 (.957) and o10 (.460) loaded on C5; c3 (.892) and c8 (.552) loaded on C4 (c8 also loaded .422 on C2); n9 loaded (.827) on C3, while n4 had its largest positive loading (.292) on C3, but also loaded (-.758) on C2; a2 loaded (.524) on C2, while a7 loaded (-.555) on C3, with its largest positive loading (.269) on C4.

**Table 3:** Principal components analysis with varimax rotation for BFI-10 Study 2d, and for all BFI-10 data combined.

	Study 2d (N = 268)					All BFI-10 studies combined (N = 1853)				
	Component					Component				
Item	1	2	3	4	5	1	2	3	4	5
e1R	**.939**	-.039	-.023	-.109	.026	**.891**	-.177	-.068	-.059	.076
e6	**.710**	-.254	**.348**	.205	.049	**.762**	**.331**	.022	.127	.100
a2	.167	-.052	.132	-.047	**.694**	-.067	**.524**	-.046	.103	.070
a7R	-.116	-.053	-.118	.062	**.819**	-.186	.098	**-.555**	.269	.063
c3R	.088	-.038	-.111	**.931**	.145	.060	-.104	-.227	**.892**	-.029
c8	-.048	-.023	.126	**.577**	-.087	.003	**.422**	.266	**.552**	.008
n4R	-.026	**.878**	-.210	.065	-.004	-.212	**-.758**	.292	.166	.056
n9	-.177	**.779**	.146	-.139	-.119	-.222	-.119	**.827**	.091	.021
o5R	-.039	-.022	**.848**	.094	.054	.065	-.016	-.103	-.017	**.957**
o10	.190	-.028	**.694**	-.031	-.033	.261	**.384**	**.307**	-.001	**.460**

e = extraversion; a = agreeableness; c = conscientiousness; n = neuroticism; o = openness. All absolute loadings > .3 are in **bold**. R = Reverse-scored items (adjusted so that all item loadings for a personality domain should be positive).

For comparison, the corresponding PCA for each BFI-10 study (studies 1a, 2a-2d) showed that the structure varied to some extent between samples (complete tables of loadings for each study are available from the authors). For Study 1a (*N* = 233) only four components were initially extracted (this was also the case for the corresponding analysis on the BFI-10X, extracted from the BFI-44). When a five-component solution was specified, e1 (.871) and e6 (.840) loaded on C1; c3 (.915) and c8 (.604) loaded on C4; n4 (.842) and n9 (.827) loaded on C2; o5 (.943) and o10 (.413) loaded on C3; a7 loaded most strongly on C5 (.968), while a2 loaded on C3 (.463).

For Study 2a (*N* = 952) five components were extracted; e1 (.880) and e6 (.734) loaded on C1; c3 (.861) and c8 (.614) loaded on C4; o5 (.958) and o10 (.448) loaded on C5.

For Study 2b (*N* = 211) five components were extracted; e1 (.920) and e6 (.790) loaded on C1; a2 (.805) and a7 (.663) loaded on C2; n4 (.811) and n9 (.766) loaded on C3; o5 (.901) and o10 (.564) loaded on C5.

For Study 2c (*N* = 189) five components were extracted; e1 (.849) and e6 (.800) loaded on C1; o5 (.928) and o10 (.590) loaded on C3.

For Study 2d (*N* = 268) five components were extracted, with all items loading on the relevant big five domain, with minimal cross-loadings (Table 3).

In summary, although the expected BFI-10 structure was clearly shown in one study, it was not consistently revealed across samples, or in the combined data. The importance of this is considered further in the Discussion.

### Convergent and discriminant validity

Inter-correlations between big five personality dimensions are shown in Table 4 for the BFI-44, BFI-10, and also the BFI-10X (the alternative version of the BFI-10, extracted from the relevant items of the BFI-44). Extraversion, agreeableness, conscientiousness, and openness all had positive inter-correlations, and all were negatively correlated with neuroticism. Inter-correlations between the BFI-44 dimensions (Table 4, top left) had a mean of .329 (all < .5), the largest being negative correlations between neuroticism and extraversion, agreeableness and conscientiousness, and a positive correlation between extraversion and openness. The same general pattern was found for the BFI-10X (Table 4, centre), for which all inter-correlations were < .4 (mean = .184), and the BFI-10 (Table 4, lower right), for which all inter-correlations were < .3 (mean = .100).

**Table 4:** Inter-correlations for the BFI-44, BFI-10X, and BFI-10.

	**BFI-44**					**BFI-10X**					**BFI-10**				
**BFI-44**	E	A	C	N	O	E	A	C	N	O	E	A	C	N	O
E	(.755)*														
A	.313	(.721)													
C	.268	.372	(.766)												
N	-.490	-.421	-.406	(.743)											
O	.409	.248	.190	-.133	(.777)										
**BFI-10X**															
E	**.840**	.311	.175	-.380	.305	(.523)									
A	.157	**.720**	.234	-.318	.112	.175	(.137)								
C	.193	.330	**.803**	-.273	.148	.106	.183	(.383)							
N	-.444	-.322	-.315	**.800**	-.183	-.331	-.230	-.180	(.444)						
O	.331	.225	.143	-.110	**.827**	.266	.104	.110	-.147	(.347)					
**BFI-10**															
E	**.799**	.321	.067	-.393	.359	**.798**	.141	.040	-.381	.333	(.647)				
A	.080	**.619**	.184	-.240	.149	.119	**.642**	.177	-.193	.144	.036	(.287)			
C	.264	.351	**.770**	-.292	.196	.193	.160	**.757**	-.212	.149	.039	.121	(.384)		
N	-.445	-.241	-.167	**.703**	-.225	-.353	-.210	-.066	**.737**	-.231	-.232	-.141	-.051	(.481)	
O	.322	.289	.044	-.168	**.766**	.260	.241	.035	-.227	**.800**	.253	.010	.023	-.089	(.410)

E = extraversion; A = agreeableness; C = conscientiousness; N = neuroticism; O = openness.

BFI-44 and BFI-10X scale inter-correlations, *N* = 798; BFI-10 scale inter-correlations, *N* = 1853; BFI-44/BFI-10, and BFI-10X/BFI-10 inter-correlations, *N* = 234–235.

*Coefficient alpha for total/combined data in parentheses.

Convergent and discriminant correlations between the BFI-44 and BFI-10X (Table 4, centre left), were very clear, with the corresponding big five dimensions all (except for agreeableness) showing correlations ≥ .8 (mean = .802), with all discriminant correlations being < .5 (mean = .252). The independent BFI-10 also showed good convergent correlations with the BFI-44 (Table 4, lower left; mean = .737), and with the extracted BFI-10X (Table 4, lower centre; mean = .752), with all convergent correlations being > .7 (except for agreeableness). Discriminant correlations for the BFI-10 were all < .4 (except the BFI-44 extraversion/BFI-10 neuroticism correlation), with most being < .3 (BFI-10/BFI-44 mean = .243; BFI-10/BFI-10X mean = .195).

### Descriptive statistics

The DF, MW, MLO and CES scales each had values of coefficient alpha > .75 in all studies. For the 5-item rMEQ values of alpha ranged .449 to .656. Means, standard deviations and values of alpha for each study are available from the authors.

Descriptive statistics for the BFI-44 are shown in Table 5. Item means showed consistency in their rankings, with highest to lowest generally being: agreeableness, openness, conscientiousness, extraversion, neuroticism. Values of Cronbach's alpha and test-retest coefficients were good, all being > .7 (except agreeableness for studies 1a and 1d). Values of Cronbach's alpha for the combined BFI-44 data are shown in Table 4 (range = .721-.777).

**Table 5:** BFI-44 descriptive statistics.

	Mean (sd)				Cronbach's alpha (mean inter-item correlation)				Test-retest coefficient†
	Study 1a	Study 1b	Study 1c	Study 1d	Study 1a	Study 1b	Study 1c	Study 1d	Study 1a
E	3.21 (.615)	3.23 (.587)	3.27 (.581)	3.15 (.619)	.778 (.307)	.720 (.248)	.770 (.294)	.741 (.262)	.770
A	3.81 (.528)	3.89 (.503)	3.81 (.527)	3.75 (.522)	.735 (.244)	.705 (.222)	.734 (.240)	.698 (.211)	.694
C	3.41 (.546)	3.51 (.581)	3.45 (.601)	3.26 (.566)	.732 (.239)	.780 (.292)	.806 (.319)	.727 (.231)	.737
N	2.92 (.581)	2.93 (.637)	2.94 (.604)	2.96 (.631)	.720 (.242)	.776 (.303)	.754 (.270)	.742 (.267)	.766
O	3.56 (.577)	3.48 (.603)	3.54 (.545)	3.35 (.583)	.785 (.271)	.807 (.294)	.766 (.253)	.737 (.224)	.738

E = extraversion; A = agreeableness; C = conscientiousness; N = neuroticism; O = openness.

Study 1a, *N* = 235. Study 1b, *N* = 184 (openness = 183). Study 1c, *N* = 172 (conscientiousness = 171).

Study 1d, *N* = 212 (neuroticism = 213).

^†^*n* = 91; all *ps* < .0005.

Descriptive statistics for the BFI-10 are shown in Table 6. Item means showed some consistency in ranking with highest to lowest generally being: openness, agreeableness, conscientiousness, extraversion, neuroticism. Values of Cronbach's alpha ranged .593-.752 for extraversion; .037-.466 for agreeableness; .251-.462 for conscientiousness; .331-.628 for neuroticism; and, .364-.525 for openness. The highest values for each domain were all reasonable for two-item scales, but there was noticeable variation between the studies, in particular with the very low value of .037 for agreeableness in Study 1a. Similarly, for the BFI-10X agreeableness dimension (i.e., the two items from the BFI-44 corresponding to the BFI-10 agreeableness items), alpha was .173. The other BFI-10X alphas for Study 1a were: extravert, .585; conscientiousness, .442; neuroticism, .406; openness, .368. Values of Cronbach's alpha for the combined BFI-10 data (range = .287-.647), and the combined BFI-10X data (range = .137-.523), are shown in Table 4; for both scales, extraversion ranked highest and agreeableness lowest.

**Table 6:** BFI-10 descriptive statistics.

	Mean (sd)					Cronbach's alpha (2-item correlation)					Test-retest†			
	S1a	S2a	S2b	S2c	S2d	S1a	S2a	S2b	S2c	S2d	S1a	S2b	S2c	mean
E	3.33 (.942)	3.23 (.990)	3.24 (.990)	3.05 (1.056)	3.41 (.999)	.752 (.603)	.593 (.423)	.704 (.546)	.704 (.550)	.675 (.511)	.803	.823	.873	.835
A	3.69 (.669)	3.66 (.843)	3.63 (.871)	3.59 (.830)	3.64 (.826)	.037 (.020)	.295 (.174)	.466 (.304)	.269 (.157)	.299 (.176)	.515	.811	.731	.705
C	3.48 (.814)	3.44 (.883)	3.28 (.847)	3.40 (.816)	3.36 (.830)	.462 (.313)	.394 (.251)	.251 (.146)	.286 (.174)	.459 (.309)	.668	.645	.706	.674
N	2.81 (.856)	3.01 (.927)	2.92 (.960)	3.05 (.926)	2.99 (.939)	.628 (.458)	.429 (.273)	.502 (.335)	.331 (.198)	.593 (.422)	.610	.749	.837	.746
O	3.81 (.803)	3.79 (.886)	3.83 (.898)	3.83 (.911)	3.94 (.849)	.438 (.283)	.364 (.225)	.382 (.238)	.447 (.291)	.525 (.358)	.698	.821	.839	.793

E = extraversion; A = agreeableness; C = conscientiousness; N = neuroticism; O = openness. S1a–S2d = Study 1a to Study 2d. Study 1a, *N* = 235 (agreeableness and conscientiousness, *N* = 234). Study 2a, *N* = 975 (agreeableness, *N* = 973; conscientiousness, *N* = 972; openness, *N* = 974). Study 2b, *N* = 213 (agreeableness and neuroticism, *N* = 212). Study 2c, *N* = 189. Study 2d, *N* = 268.

^†^S1a, *n* = 90/91; S2b, *n* = 78/79; S2c, *n* = 91. All *ps* < .0005.

BFI-10 test-retest coefficients were generally good, ranging .803-.873 for extraversion, .515-.811 for agreeableness, .645-.706 for conscientiousness, .610-.837 for neuroticism, and .698-.839 for openness (Table 6). Again there was some variation between studies, but all coefficients were > .6, except for agreeableness in Study 1a. Test-retest coefficients for the BFI-10X (Study 1a; *n* = 91) were: .597 (extraversion); .546 (agreeableness); .595 (conscientiousness); .631 (neuroticism); .571 (openness).

Test-retest coefficients for each BFI-10 item, and the alpha coefficients for only those participants who completed both test (T1) and retest (T2) sessions, are given in Table 7. Test-retest correlations for each BFI-10 item showed generally consistent coefficients across the three studies, although the values for agreeableness (and also openness and neuroticism) were somewhat lower in Study 1a. The alpha values for T1 and T2 sessions were generally similar within each study, suggesting that the variation between studies was related to sample differences. However, there were some larger variations between T1 and T2 values, in particular for agreeableness in Study 2c (and to a lesser extent in Study 1a), which may suggest situational influences on item responses.

**Table 7:** BFI-10 item retest coefficients, and alpha coefficients for test and retest sessions.

	Study 1a				Study 2b				Study 2c			
	T1	T2	Retest coefficients		T1	T2	Retest coefficients		T1	T2	Retest coefficients	
	alpha (2-item correlation)	alpha (2-item correlation)	item 1 (item 2)	overall	alpha (2-item correlation)	alpha (2-item correlation)	item 1 (item 2)	overall	alpha (2-item correlation)	alpha (2-item correlation)	item 1 (item 2)	overall
E	.788 (.651)	.687 (.523)	.788 (.647)	.803	.722 (.572)	.795 (.661)	.783 (.763)	.823	.762 (.630)	.683 (.525)	.799 (.774)	.873
A	.122 (.066)	.243 (.142)	.386 (.504)	.515	.439 (.283)	.498 (.334)	.760 (.771)	.811	.458 (.299)	.189 (.104)	.588 (.684)	.731
C	.356 (.223)	.272 (.162)	.703 (.554)	.668	.138 (.075)	-.090 (-.043)	.652 (.506)	.645	.329 (.199)	.293 (.181)	.710 (.596)	.706
N	.626 (.457)	.639 (.470)	.568 (.456)	.610	.605 (.433)	.716 (.559)	.579 (.716)	.749	.532 (.363)	.494 (.330)	.710 (.755)	.837
O	.329 (.198)	.393 (.246)	.599 (.530)	.698	.291 (.173)	.324 (.196)	.857 (.645)	.821	.551 (.381)	.499 (.336)	.826 (.735)	.839

E = extraversion; A = agreeableness; C = conscientiousness; N = neuroticism; O = openness. T1 = test 1; T2 = test 2. Study 1a, *n* = 90/91; Study 2b, *n* = 78/79; Study 2c, *n* = 91.

### Age and gender

Regression analysis was undertaken separately for each big five domain, with age and gender (coded 0 = male; 1 = female) as predictor variables (cases missing any big five domain, or with missing age or gender information, were excluded). For the combined BFI-44 data (studies 1a-1d), *N* = 773; 302 male, 471 female, aged 18–67 (74.4% aged 30/younger); mean age = 27.10 (sd = 7.21); male mean = 28.63 (sd = 7.35); female mean = 26.12 (sd = 6.95); *t* = 4.787, *p* < .0005. For the combined BFI-10 data (studies 1a, and 2a-2d), *N* = 1823; 540 male, 1283 female, aged 18–82 (82.3% aged 30/younger); mean age = 24.76 (sd = 8.42); male mean = 27.37 (sd = 8.98); female mean = 23.66 (sd = 7.92); *t* = 8.343, *p* < .0005.

As shown in Table 8, for both the BFI-44 and the BFI-10, there were trends (although not always reaching significance), for more extraversion, agreeableness, neuroticism and openness in females, and for older age to be associated with more agreeableness and conscientiousness, but less neuroticism and openness.

**Table 8:** Age and gender as predictors for the big five personality domains.

Scale / study	Dependent variable	*R*	Adjusted *R^2^*	*F*	Gender *β*	Age *β*
All BFI-44	Extraversion	.086	.005	2.879†	.086*	.032
*N* = 773	Agreeableness	.120	.012	5.643**	.096**	.091*
(df: 2, 770)	Conscientiousness	.239	.054	23.244***	-.003	.238***
	Neuroticism	.137	.016	7.334**	.050	-.119**
	Openness	.171	.027	11.604***	.162***	-.033
All BFI-10	Extraversion	.097	.008	8.599***	.089***	-.025
*N* = 1823	Agreeableness	.108	.011	10.679***	.094***	.074**
(df: 2, 1820)	Conscientiousness	.249	.061	60.112***	-.036	.239***
	Neuroticism	.080	.005	5.876**	.072**	-.023
	Openness	.175	.029	28.665***	.092***	-.131***

^†^*p* ≤ .10;

*p ≤ .05;

**p ≤ .01;

***p ≤ .0005.

### Nomological network

Across all of the studies, the BFI-44 and BFI-10 showed similar, generally consistent correlations with chronotype (morningness-eveningness preference), as assessed with the rMEQ (Table 9). In particular morningness (higher rMEQ scores) showed a consistent positive correlation with conscientiousness (*rs* ranging .127 to .331; significant in all studies except Study 1c). Morningness also showed significant positive correlations with agreeableness in three studies, while correlations with extraversion, neuroticism and openness were variable though generally weak. Overall, these results are consistent with the findings of Tsaousis' [32] meta-analysis, which showed conscientiousness to be the strongest personality correlate of chronotype, with agreeableness ranking second.

**Table 9:** Correlations between the big five personality domains and chronotype.

	Chronotype (rMEQ)									
	BFI-44†				BFI-10†					Tsaousis [32]‡
	S1a	S1b	S1c	S1d	S1a	S2a	S2b	S2c	S2d	
E	.048 (225)	.056 (167)	.196* (165)	.105 (202)	.025 (225)	.019 (938)	.024 (213)	.002 (189)	.067 (268)	**-.06**
A	.022 (225)	.215** (167)	.205** (165)	-.021 (202)	.002 (224)	.057 (936)	.214** (212)	.087 (189)	.004 (268)	**.13**
C	.155* (225)	.203** (167)	.127 (164)	.194** (202)	.149* (224)	.204*** (934)	.218** (213)	.331*** (189)	.214*** (268)	**.29**
N	.015 (225)	-.043 (167)	-.143 (165)	-.096 (203)	-.005 (225)	-.004 (937)	.102 (212)	-.013 (189)	.002 (268)	**-.07**
O	.017 (225)	.054 (167)	.146 (165)	-.046 (202)	.008 (225)	.013 (937)	-.159* (213)	.051 (189)	.178** (268)	**-.09**

E = extraversion; A = agreeableness; C = conscientiousness; N = neuroticism; O = openness; rMEQ = reduced Morningness-Eveningness Questionnaire.

^†^Partial correlations, controlling for age; *N*-values in parentheses. S1a–S2d = Study 1a–Study 2d.

*p ≤ .05;

**p ≤ .01;

***p ≤ .0005.

^‡^Results from Tsaousis' [32] meta-analysis.

Mindfulness (the MLO scale) had significant negative correlations with neuroticism in all studies, and significant positive correlations with extraversion and conscientiousness in 2/3 studies (Table 10). Also, BFI-44 Study 1d showed significant positive correlations with agreeableness and openness, which did not reach significance in the BFI-10 studies. Overall, the observed coefficients show reasonably close correspondence with those from Giluk's [34] meta-analysis: all correlations were positive, except the (stronger) negative correlations with neuroticism.

**Table 10:** Correlations between the big five personality domains and daydreaming frequency, mind wandering, attention-related cognitive errors, and mindfulness.

	Daydreaming Frequency†				Mind Wandering†				CES†	Mindfulness†			
	S1b	S2a	S2b	S2d	S1c	S2a	S2c	S2d	S2a	S1d	S2a	S2d	Giluk [34]‡
E	-.250** (180)	-.109* (329)	-.087 (213)	-.069 (268)	-.266*** (172)	-.141 (192)	.031 (189)	-.071 (268)	-.171** (258)	.287*** (209)	.156* (185)	.056 (174)	**.12**
A	-.185* (180)	-.031 (328)	-.040 (212)	-.107 (268)	-.266*** (172)	-.008 (191)	-.085 (189)	-.120* (268)	.021 (259)	.375*** (209)	.128 (184)	.088 (174)	**.22**
C	-.376*** (180)	-.226*** (326)	-.205** (213)	-.326*** (268)	-.581*** (171)	-.376*** (190)	-.308*** (189)	-.492*** (268)	-.192** (260)	.365*** (209)	.121 (185)	.252** (174)	**.32**
N	.389*** (180)	.073 (328)	.255*** (212)	.208** (268)	.435*** (172)	.274*** (190)	.224** (189)	.222*** (268)	.199** (260)	-.385*** (210)	-.386*** (185)	-.193* (174)	**-.45**
O	.013 (180)	.112* (328)	.180** (213)	.054 (268)	-.105 (172)	-.073 (189)	-.034 (189)	.005 (268)	-.092 (260)	.211** (209)	.121 (185)	.109 (174)	**.15**

E = extraversion; A = agreeableness; C = conscientiousness; N = neuroticism; O = openness.

^†^Partial correlations, controlling for age; *N*-values in parentheses. S1a–S2d = Study 1a–Study 2d. CES = The Attention-related Cognitive Errors Scale.

*p ≤ .05;

**p ≤ .01;

***p ≤ .0005.

^‡^Results from Giluk's [34] meta-analysis for mindfulness.

The Attention-related Cognitive Errors Scale (CES) showed significant negative correlations with extraversion and conscientiousness, and a significant positive correlation with neuroticism (Table 10). The CES correlates strongly with the Cognitive Failures Questionnaire [60, 61] which also positively correlates with neuroticism [62].

Both mind wandering (the MW scale) and daydreaming frequency (the DF scale) had significant negative correlations with conscientiousness in all studies (Table 10; cf. [40]). Also, they both had significant positive correlations with neuroticism (except DF in Study 2a). Coefficients with the BFI-44 were generally stronger than with the BFI-10. Furthermore, in BFI-44 studies 1b and 1c, DF and MW had significant negative correlations with extraversion, which were not significant with the BFI-10 (except for DF in Study 2a). Three studies (one with the BFI-10) also showed significant negative correlations with agreeableness. However, DF was significantly positively correlated with openness in two of the BFI-10 studies; MW did not show any significant correlations with openness.

As the participants in Study 2d also completed the MLO scale (measure of mindfulness) 4–6 weeks after completing the BFI-10, linear regression analysis was undertaken for each big five domain, to test for significant predictors of the later MLO scores. For conscientiousness, *R* = .252, adjusted *R²* = .058; *F*(1,172) = 11.647; *β* = .252, *p* = .001. For neuroticism, *R* = .196, adjusted *R²* = .033; *F*(1,172) = 6.897; *β* = -.196, *p* = .009. Analyses for openness, extraversion, and agreeableness were all non-significant (*ps* > .1).

### Exploratory study of associations with Big Five facets

Further, exploratory correlational analysis (Table 11) was done with the big five facets (two for each domain) that can be assessed with 35 of the BFI-44 items (for details, see [5]). Across the four BFI-44 studies, chronotype only showed consistent significant correlations (all *rs* > .1) with Self-discipline (example items: "Perseveres until the task is finished" / "Is easily distracted", reverse-scored); more Self-discipline was associated with more morningness. Self-discipline and the other conscientiousness facet of Order (items: "Tends to be disorganised", reverse-scored / "Can be somewhat careless", reverse-scored) were also consistently correlated with mind wandering, daydreaming frequency and mindfulness, as were several other facets, in particular the neuroticism facets of anxiety and depression.

**Table 11:** Correlations between big five personality facets and chronotype, daydreaming frequency, mind wandering, and mindfulness.

	Chronotype (rMEQ)†				Daydreaming Frequency†	Mind Wandering†	Mindfulness†
Domains/Facets	Study 1a	Study 1b	Study 1c	Study 1d	(Study 1b)	(Study 1c)	(Study 1d)
Extraversion							
Assertiveness	-.022	-.001	.150	.084	-.277***	-.250**	.220**
Activity	.138*	.079	.173*	.118	-.138	-.159*	.337***
Agreeableness							
Altruism	.030	.192*	.212**	-.055	-.105	-.257**	.311***
Compliance	-.035	.103	.145	.045	-.149*	-.169*	.342***
Conscientiousness							
Order	.105	.091	-.037	.165*	-.216**	-.327***	.185**
Self-discipline	.136*	.195*	.172*	.174*	-.386***	-.596***	.332***
Neuroticism							
Anxiety	.011	-.026	-.111	-.081	.312***	.399***	-.351***
Depression	-.009	-.039	-.211**	-.091	.339***	.348***	-.295***
Openness							
Openness to aesthetics	.023	.026	.019	.033	.076	-.072	.162*
Openness to ideas	-.018	.002	.217**	-.076	-.014	-.128	.161*

^†^Partial correlations, controlling for age; *N*-values as for Tables 9 and 10; rMEQ = reduced Morningness-Eveningness Questionnaire.

*p ≤ .05;

**p ≤ .01;

***p ≤ .0005.

Previous research [39] found that sleep quality and positive affect were mediators in the associations between chronotype (rMEQ; predictor) and mind wandering (MW), and chronotype (rMEQ; predictor) and daydreaming frequency (DF). As Self-discipline was found to consistently correlate with rMEQ, MW, DF, and MLO, it was tested whether Self-discipline might also be a mediator in these associations (with age and gender as control variables). Following the procedures suggested by Preacher and Hayes [63, 64]: *path a* represents the effect of the predictor (independent variable/IV) on the mediator; *path b* represents the mediator's direct effect on the criterion (dependent variable/DV), while controlling for the IV; *path c* represents the total effect of the IV on the DV, when the proposed mediator is excluded (this does not need to be significant for the existence of a mediation effect); *path c'* represents the IV's direct effect on the DV (controlling for the mediator); and, *path ab* represents the indirect effect of the IV on the DV, through the mediator. This indirect effect was tested with a non-parametric bootstrapping procedure [63, 64], in which 5000 re-samples from the data established 95% confidence intervals, with the exclusion of zero indicating a significant mediation effect.

As shown in Table 12, Self-discipline was a significant mediator in the associations between chronotype and: daydreaming frequency (Model 1), mind wandering (Model 2), and mindfulness (Model 3). Chronotype retained a significant direct effect for mindfulness, and a marginally significant direct effect for daydreaming frequency, suggesting partial mediation by Self-discipline in these models. Chronotype did not have significant total or direct effects for mind wandering.

**Table 12:** Self-discipline as a mediator between chronotype and daydreaming frequency, mind wandering, and mindfulness.

Model 1: Predictor/IV = Chronotype (rMEQ); Criterion/DV = Daydreaming Frequency (DF)							
	IV	mediator	DV	IV *β* (*t*)	Mediator *β* (*t*)	Age *β* (*t*)	Gender *β* (*t*)
*Path c*	rMEQ	-	DF	-.196* (-2.468)	-	.054 (.684)	.225** (2.833)
*Path a*	rMEQ	Self-discipline	-	.221** (2.789)	-	.089 (1.133)	-.042 (-.531)
*Paths b and c'*	rMEQ	Self-discipline	DF	-.128† (-1.671)	-.338*** (-4.543)	.087 (1.153)	.215** (2.853)
Final Model: *R* = .421; adjusted *R²* = .156; *F*(4, 157) = 8.445***							
(Unstandardised) indirect effect (*path ab*): mean estimate = -.1824; bias corrected 95% confidence interval = -.3874 to -.0396							
Model 2: Predictor/IV = Chronotype (rMEQ); Criterion/DV = Mind Wandering (MW)							
	IV	mediator	DV	IV *β* (*t*)	Mediator *β* (*t*)	Age *β* (*t*)	Gender *β* (*t*)
*Path c*	rMEQ	-	MW	-.154 (-1.950)	-	-.148 (-1.835)	-.059 (-.739)
*Path a*	rMEQ	Self-discipline	-	.170* (2.171)	-	.189* (2.365)	.001 (.017)
*Paths b and c'*	rMEQ	Self-discipline	MW	-.056 (-.866)	-.603*** (-9.293)	-.032 (-.486)	-.060 (-.936)
Final Model: *R* = .626; adjusted *R²* = .376; *F*(4, 156) = 25.132***							
(Unstandardised) indirect effect (*path ab*): mean estimate = -.2211; bias corrected 95% confidence interval = -.4804 to -.0068							
Model 3: Predictor/IV = Chronotype (rMEQ); Criterion/DV = Mindfulness (MLO)							
	IV	mediator	DV	IV *β* (*t*)	Mediator *β* (*t*)	Age *β* (*t*)	Gender *β* (*t*)
*Path c*	rMEQ	-	MLO	.226** (3.168)	-	.118 (1.547)	-.011 (-.140)
*Path a*	rMEQ	Self-discipline	-	.208** (2.894)	-	.029 (.382)	-.058 (-.762)
*Paths b and c'*	rMEQ	Self-discipline	MLO	.161* (2.299)	.296*** (4.245)	.107 (1.458)	.010 (.134)
Final model: *R* = .396; adjusted *R²* = .138; *F*(4, 184) = 8.555***							
(Unstandardised) indirect effect (*path ab*): mean estimate = .1908; bias corrected 95% confidence interval = .0648 to .3900							

DF = Daydreaming Frequency; MW = Mind Wandering; MLO = Mindful Attention Awareness Scale-Lapses Only; rMEQ = reduced Morningness-Eveningness Questionnaire.

^†^*p* ≤ .10;

*p ≤ .05;

**p ≤ .01;

***p ≤ .0005.

## Discussion

### Scale structure

PCA for the BFI-44 showed that most items loaded strongly or predominantly on their corresponding component (at least 50% of items loading at least > .3). Although the observed scale structure was not as strong/clear as desirable, or as reported in other studies with Chinese samples (e.g., [12, 13] for the NEO-PI-R, and [14] for the IPIP), the reasons and implications need consideration. It might be that translations of some items could be revised. In addition, many of the poorer-loading items were reverse-scored, which might produce some culturally specific effects in responding [15, 28]. Also, there could be some cultural differences in the meaning, interpretation or relevance of some items. In this regard, it is noteworthy that three of the openness items (30, 41, and 44) loaded strongly on a sixth component. Throughout the development of the big five model there has been particular disagreement regarding the definition of the openness/intellect domain, and cross-cultural studies, including studies in China, have shown less consistency in producing a clear openness/intellect factor [3, 11, 65]. Use of a translated BFI-44 constitutes an imposed etic, in which a personality structure from another culture is imposed, rather than seeking to discover an indigenous structure [9, 11]. However, differences found in cross-cultural research may be due to cultural differences, translation-related differences in the instrument, and/or sample differences. Further cross-cultural research involving bi-lingual participants would help to illuminate translation issues and culture differences [9]. Cross-language generality may also be underestimated due to mistranslations that remain undetected in research using monolingual samples [3].

Although additional factors (beyond the big five), indicated in some cross-cultural studies, have not always been consistently replicated [2], some evidence suggests that including a sixth factor (of Honesty/Propriety) may improve validity for some criterion measures and have more cross-cultural replicability [8]. In Chinese research, Cheung et al. [65] supported a six-factor model, while Zhou et al.'s [66] lexical study suggested a seven-factor emic structure. Further research is needed on these proposed personality structures, and before firm conclusions are reached about personality structure within a group, repeated testing on large samples may be prudent. Methods of exploratory and confirmatory factor analysis are more effective with large samples [55]; Costello and Osborne [54] found that even with a subject/item ratio of 20:1, 30% of solutions in simulated studies failed to produce a known population factor structure. Therefore, further testing of the current, and/or any revised versions of the BFI-44, should be undertaken, with large, comparable samples (including a more balanced gender ratio and specific age range coverage), if a stable, replicable structure is to be established. For example, the 29-item BFI (in traditional Chinese characters) developed by Leung et al. [15] from a sample of 480 smokers and ex-smokers (mean age = 40.6; 83.8% male) was not strongly supported in the current research involving part-time and full-time students (*N* = 798; mean age = 27.11; 38.6% male). Only one index of model fit (RMSEA) was within an acceptable range for the 29-item BFI, but this was also the case for the full BFI-44.

Some previous studies have reported clear scale structures for the BFI-10 and Ten-Item Personality Inventory (TIPI; e.g., [10, 22]). Likewise, in Study 2d of the current research, PCA of the BFI-10 clearly showed the expected big five structure, with each item loading strongly on its corresponding component. However, for the other BFI-10 studies (and the combined data), only the extraversion and openness items clearly loaded on the same component in each study, while the items for the other big five dimensions showed less consistency. Variation in demographic characteristics between samples might have had some influence (Study 2d had the most homogeneous sample in terms of age, all being 18–21), which could be investigated in further research. However, while it might be that some of the BFI-10 item translations could be improved, the significance of the scale structure of the BFI-10, when compared with other evidence for its validity, is not clear; as Gosling [57] states: "… Criteria like alpha and clean factor structures are only meaningful to the extent they reflect improved validity. In cases like the TIPI (using a few items to measure broad domains), they don’t."

In summary, PCA of the BFI-44 and BFI-10 indicated the big five structure, although not as clearly or consistently as would be preferred. This might be improved by revisions to some items, but further testing with large samples is needed for a stable, replicable structure to be established.

### Convergent and discriminant validity

Inter-correlations between BFI-44 dimensions were generally low, the largest being between .4 and .5, with a mean of .329. These values are somewhat higher, though comparable to the mean of around .2, and high of around .3, reported in other studies [2, 9, 10]. All inter-correlations were < .3 for the BFI-10, with a mean of .100, which is very close to the mean of .11 reported by Rammstedt and John [10]. All inter-correlations were < .4, with a mean of .184 for the BFI-10X (extracted from the BFI-44). Extraversion, agreeableness, conscientiousness, and openness were all positively inter-correlated, and each negatively correlated with neuroticism (cf. [9, 10, 15]). The strongest inter-correlations were a positive correlation between extraversion and openness, and negative correlations between neuroticism and extraversion, agreeableness and conscientiousness (cf. [9, 19]).

There were clear convergent and discriminant correlations between the BFI-44 and BFI-10, and also between the BFI-10 and BFI-10X, where, in all cases, each dimension on each scale correlated most strongly with the corresponding dimension on the other scale. Nearly all convergent correlations were > .7, and discriminant correlations < .4 (most being < .3). As the BFI-44 and BFI-10 in the current studies were independent translations, the 10-item scale extracted from the BFI-44 (the BFI-10X) is also available as an alternative version, which allows for use of equivalent forms. However, the Chinese BFI-44 and BFI-10 were both translated from the same original English-language scale, so further research should include comparisons with other measures of the big five personality dimensions, such as the NEO-PI-R, NEO-FFI, or the IPIP.

### Mean rankings, age and gender

The mean rankings of the big five dimensions were very consistent across the four BFI-44 studies, with highest to lowest generally being agreeableness (A), openness (O), conscientiousness (C), extraversion (E), and neuroticism (N). Highest to lowest rankings of A/O, C, E, N were reported by Benet-Martinez and John [9] for both English and Spanish versions of the BFI-44, and by Zheng et al. [14] for Chinese-language IPIP scales with their sample of heterosexual and homosexual Chinese, while Leung et al. [15], with their reduced, 29-item BFI, found highest to lowest of C, A, E, O, N, in their sample of Chinese smokers and ex-smokers. The mean rankings for the BFI-10 were generally consistent across studies, and similar to the rankings for the BFI-44, with highest to lowest generally being O, A, C, E, N. Credé et al. [20] reported highest to lowest of A, O, C, E, N, for the BFI-10, in a student sample, and C, A, O, N, E, in a sample of workers.

Regarding age differences, the results of the current research are consistent with previous findings (e.g., [6, 28, 29, 30]): age was positively associated with agreeableness and conscientiousness, and negatively associated with neuroticism and openness. Likewise, in a Chinese sample of mostly psychiatric patients, Yang et al. [13], using the NEO-PI-R, found that age positively correlated with conscientiousness and agreeableness, and negatively correlated with openness and neuroticism.

Regarding gender differences, the current research with Chinese participants found that females generally scored higher for agreeableness, neuroticism, extraversion, and openness, as has been found in many other countries [31]. Likewise, in previous Chinese research, Yang et al. [13] found that females scored higher for agreeableness, and Zhang and Huang [67], using the NEO-FFI with university students, found that females scored higher for openness and agreeableness. With their 29-item BFI, Leung et al. [15] found (in 368 male smokers/ex-smokers compared with 71 females) significantly higher scores in females for neuroticism, but significantly lower scores for openness.

### Internal consistency

The Chinese version of the BFI-44 showed acceptable/good values of internal consistency (coefficient alpha) in all studies. Values ranged .698-.807; agreeableness had the lowest ranking value in 3/4 studies. These values are generally comparable to those reported in other studies of the BFI-44. Alphas for the English BFI-44 usually range .75-.90, and agreeableness often ranks lower than the other domains [2, 3]; for example, Benet-Martinez and John [9] found that agreeableness had the lowest rank in both English and Spanish versions of the BFI-44 (< .7 for the Spanish version). The current alpha values are also comparable to those reported for other Chinese scales, including Leung et al.'s [15] 29-item BFI with alphas ranging .69 (agreeableness) to .81 (neuroticism); also, Zhang and Huang [67] found alpha values ranging .56 (openness) to .75 (neuroticism), for a Chinese translation of the NEO-FFI.

The Chinese version of the BFI-10 had alpha values ranging .593-.752 for extraversion; .037-.466 for agreeableness; .251-.462 for conscientiousness; .331-.628 for neuroticism; and .364-.525 for openness. The highest values for each domain were all reasonable for two-item scales (all > .45), and comparable to alpha values reported in other studies using the BFI-10, or Ten-Item Personality Inventory (TIPI), many of which have also reported the highest value for extraversion, and lowest for agreeableness, as generally found in the current studies. For example, Thalmayer et al. [8] reported BFI-10 alphas ranging .43 (agreeableness) to .72 (extraversion); Credé et al. [20] reported BFI-10 alphas ranging .37 (agreeableness) to .65 (extraversion); and Gosling et al. [16] reported TIPI alphas ranging .40 (agreeableness) to .73 (emotional stability).

However, the lower values of alpha in the current studies were < .3, with a low of .037 for agreeableness in Study 1a, which might indicate poor quality data, perhaps related to careless responding in that sample [68], at least for that subscale (the other domains had alphas > .4 in Study 1a). Some lower values of alpha have also been reported for the TIPI; for example, Woods and Hampson [17] reported .25 for openness, and Renau et al. [23] reported .21 for the agreeableness subscale of their Spanish TIPI. Low values have also been found for longer scales in some samples. For example, Oshio et al. [24] reported an alpha of .14 for the Values facet of the Openness dimension of the Japanese NEO-PI-R.

There has been some discussion about the importance of values of Cronbach's alpha, particularly in short scales such as the BFI-10 and TIPI (e.g., [8, 16, 57]). There is a trade-off between internal consistency and scale length (and so, administration time), although high internal consistency might indicate redundancy and narrowness in the scale [56]. Furthermore, McCrae et al. ([68]; see also [69]) found that retest reliability (for the NEO facet scales) significantly predicted validity criteria (including heritability and cross-observer ratings), but internal consistency (alpha) did not. Item heterogeneity (i.e., how many aspects of a trait are covered by the items in a scale) diminishes alpha, but not test-retest reliability: "… specific variance in an item is not shared by other items in the scale, so it detracts from alpha. However, in retest designs, the same items, with the same specific variance, are readministered, and they may elicit the same response. … Item-specific variance differentiates retest from internal consistency reliability, and thus may account for the superiority of the former in predicting scale validity" [69, p.2-3].

### Test-retest

The BFI-44 showed good 5–6 week test-retest reliability (Study 1a), with coefficients ranging .694 (agreeableness) to .770 (extraversion). These values are comparable to BFI-44 test-retest coefficients reported in other studies; for example, Gosling et al. [16] reported 2-week retest coefficients ranging .76 (agreeableness and conscientiousness) to .83 (neuroticism). Three-month test-retest coefficients for the English BFI-44 usually range .80-.90 [2, 3].

For the BFI-10, generally good 5–6 week test-retest coefficients were shown in studies 1a, 2b, and 2c, with ranges of: .803-.873 (extraversion); .515-.811 (agreeableness); .645-.706 (conscientiousness); .610-.837 (neuroticism); and .698-.839 (openness). These coefficients are comparable to those found in other studies. For example, Rammstedt and John [10] reported BFI-10 retest coefficients ranging .65 (openness) to .79 (extraversion) for the English language BFI-10 (8-week interval), and a range of .66 (agreeableness) to .87 (extraversion) for the German version (6-week interval). For the TIPI, Gosling et al. [16] reported 6-week retest coefficients ranging .62 (openness) to .77 (extraversion). It is notable that extraversion was the strongest dimension in these studies. A meta-analysis of test-retest reliability for the big five personality domains [70] found that extraversion showed the highest reliability while agreeableness had the lowest. In the current research, extraversion had the highest retest coefficients in all studies (BFI-44 and BFI-10), while agreeableness was lowest for the BFI-44, and the BFI-10 in Study 1a (conscientiousness was lowest in BFI-10 studies 2b and 2c). Agreeableness ratings may be more susceptible to situational influences [70], which may have had a bearing on, for example, the relatively low test-retest reliability for agreeableness in Study 1a. Given the relatively weaker psychometric properties of the BFI-10 agreeableness subscale, an optional extra agreeableness item can be included (see S1 Appendix; cf. [10])

In view of the above cited arguments and evidence for test-retest reliability being more strongly associated with validity than is coefficient alpha [8, 68, 69], the current findings of generally good test-retest reliability bode well for the Chinese BFI-44 and BFI-10 scales used in the current studies. As McCrae [69, p.12] states "… When retest reliability is high, random error is low, and it is hardly surprising that validity is higher."

### Nomological network

As discussed above, both the BFI-44 and the BFI-10 showed associations with age and gender that have been found in other Western and Asian studies. Other evidence for external/criterion-related validity was shown in the positive correlation between conscientiousness and morningness which was consistently found (significantly in all studies except BFI-44 Study 1c), replicating the finding that conscientiousness is the strongest personality correlate of chronotype [32, 33]. Also, although Hsu et al. [71], for example, found a positive association between morningness and extraversion, this was found in only one of the current studies, while a meta-analysis found a small negative correlation (*r* = -.06; [32]). As Hsu et al. [71] suggest, such inconsistencies may possibly reflect culture differences, but they may also be at least partly due to the personality scale and theoretical approach used, in addition to variation in samples [32]. For example, Hsu et al. [71], with a sample of nearly 3000 Taiwanese undergraduates, used a Chinese version of the Maudsley Personality Inventory (MPI), rather than one of the big five scales (they did not assess the big five domains of conscientiousness, agreeableness or openness).

Personality correlates of mindfulness (the MLO scale) showed correspondence with the findings from Giluk's [34] meta-analysis: mindfulness was negatively correlated with neuroticism in all studies, while positive correlations were found with all of the other personality dimensions. There was some evidence of attenuation of coefficients with the BFI-10, as significant positive correlations between mindfulness and extraversion, agreeableness, conscientiousness and openness were found with the BFI-44 in Study 1d, but these correlations were generally weaker and not consistently significant in the BFI-10 studies. Such attenuation of correlations can occur when using shorter measures of a construct, which make less fine-grained distinctions between respondents [17, 20]. However, some evidence for predictive validity (the 'acid test' for short scales [8, 56]) was shown in BFI-10 Study 2d, in which conscientiousness and neuroticism were significant predictors of mindfulness.

Regarding mind wandering (the MW scale) and daydreaming frequency (the DF scale), the observed negative correlations between conscientiousness and both MW and DF, in all studies, is consistent with the findings of Jackson and Balota [40]. Also, MW and DF were significantly positively correlated with neuroticism (including the facets of anxiety and depression) in nearly all studies, consistent with the widely reported association between mind wandering/daydreaming frequency and negative affect (e.g., [39, 42, 43, 44]). More inconsistent correlations were found with extraversion (significantly negative in three studies), and agreeableness (significantly negative in three studies). Some of this inconsistency may again be due to attenuation with the BFI-10, but it is noteworthy that positive correlations between DF and openness were only found in two BFI-10 studies. Such evidence from the BFI-10 needs to be replicated with longer personality scales, to establish whether the associations may be with the general domain or only particular facets [69]. However, an association with openness has previously been reported. Zhiyan and Singer [72] investigated big five correlates of three 'styles of daydreaming' identified from second-order factor analysis of responses to the Imaginal Processes Inventory [49, 73]. They found that the 'positive-constructive' style (frequent, vivid, and generally enjoyable daydreams) is most strongly associated with openness; the 'guilty-dysphoric' style (mixed heroic/achievement and failure/guilt daydreams) is most strongly associated with neuroticism; and the 'poor attentional-control' style (more mind wandering, more experience of boredom, and less sense of control) is most strongly associated with less conscientiousness (and more neuroticism). The current research did not assess these higher-order 'styles of daydreaming' so direct comparisons cannot be made, but the results have some correspondence, on the assumption that MW is more closely related to 'poor attention control', while DF may be more related to 'positive-constructive' and 'guilty-dysphoric' daydreaming. Further research could seek to replicate Zhiyan and Singer's [72] findings, and perhaps also investigate big five facet-level associations with the content of mind wandering/daydreaming episodes, including those more associated with positive affect [74, 75].

Overall, a consistent pattern of personality correlates was found for chronotype, mindfulness and the frequency of mind wandering/daydreaming, substantially in accord with previous findings. In addition, the exploratory study of associations with facets of the big five dimensions found that the 'self-discipline' facet of conscientiousness was a common correlate. Furthermore, self-discipline was found to mediate the relationships between chronotype (predictor) and mind wandering, daydreaming frequency, and mindfulness. Such findings show the value of more fined-grained analysis, and suggest that further research at the facet level may be insightful, possibly indicating mechanisms underlying associations between aspects of personality and cognition [72, 76].

### Limitations and future research

The current research involved a series of eight surveys which allowed for testing of the replicability/consistency of the findings for the BFI-44 and the independently translated BFI-10. However, direct comparisons between the BFI-44 and BFI-10 were limited to only Study 1a, which involved the combination of these scales into one 54-item scale. Although other studies have also analysed sub-groups of items from longer scales (e.g., [10] in the development of the BFI-10, and [14] for 100-item and 50-item IPIP scales), it might be that responses to items can vary to some extent relative to whether they are part of a shorter or longer scale [77]. Also, longer scales may induce more boredom, fatigue, frustration, and/or mistrust (e.g., from respondents' perceptions of redundant items), and may produce careless responding at similarly worded items [16, 20, 78]. This may have been so in Study 1a, as the BFI-10 is similarly worded to the corresponding items of the BFI-44, and this may have influenced responses to some items more than others. Although the results for dimension means, alphas, test-retest coefficients, etc, in Study 1a were mostly comparable to those found in the other current studies, the BFI-10 agreeableness subscale had a very low value of alpha.

So, future research comparing these BFI-44 and BFI-10 scales should present them separately, in different test sessions (or separated by other scales if within the same test session). Also, more extensive testing of convergent/discriminant validity should be done with other big five measures. Other evidence for validity, such as from peer ratings, and more extensive testing of predictive validity (including other kinds of non-questionnaire measurement, such as GPA; cf. [8]), would also be useful, as would further testing of reliability (e.g., test-retest over different intervals). Also, both scales may potentially benefit from revisions to some item translations, perhaps especially for reverse-scored items [15]. Finally, although the eight surveys in the current research involved a relatively large sample (total *N* = 2,496), the majority of these were students aged 18–30, although the demographics varied between studies. The establishment of reliable, valid normative data, and of stable, replicable scale structures, may require research with much larger samples, with different sub-groups appropriately represented. This would also allow for comprehensive investigation of any interactions between age and gender for personality changes over the lifespan (cf. [28, 30]).

### Conclusion

The current series of studies has contributed to Chinese-language personality research by detailing the psychometric properties of Chinese translations of the BFI-44 and BFI-10. Overall, there was good convergent and discriminant validity, good test-retest reliability, and expected associations with age, gender and other criterion variables. While further research may reveal important, replicable culture differences in personality structure, much international research, including in China (e.g., [13, 14, 15, 67]), continues within the big five framework, which can facilitate the comparison of findings from different countries/cultures [19]. The BFI scales offer appropriate measures, freely available for non-commercial use. While short scales like the BFI-10 save time, and may partly off-set their poorer psychometric properties by reducing participant frustration or boredom, and the careless responding that this may produce [16, 78], these advantages must be weighed against limitations, including the reduced content coverage, and attenuation of external associations [18, 20]. Consequently, we would echo the view of Gosling et al. [16] and Rammstedt and John ([10]; see also [17, 20, 78]), that the use of very short personality measures is not to be encouraged, but that these scales may be useful when there is limited time available for the research, or personality is not the main focus.

## Supporting Information

S1 Appendix (DOC)
