# Supplementary material for: Psychometric Evaluation of Chinese-Language 44-Item and 10-Item Big Five Personality Inventories, Including Correlations with Chronotype, Mindfulness and Mind Wandering
Source: PLoS One. 2016 Feb 26;11(2):e0149963. doi: 10.1371/journal.pone.0149963 (PMC4769279; doi:10.1371/journal.pone.0149963)
Supplement: S1 Appendix — (DOC) [file pone.0149963.s001.doc]

**Appendix - Chinese-language BFI-10**

下面这些句子从多大程度上能够描述你的性格？

非常 有点 既不同意 有点 非常

不同意 不同意 也不反对 同意 同意

我认为我：...

1)... 话不多 (1) (2) (3) (4) (5)

2)...或者总体而言是 (1) (2) (3) (4) (5)

信任他人的

3)...懒惰 (1) (2) (3) (4) (5)

4)... 抗压能力强，

容易放松 (1) (2) (3) (4) (5)

5)... 对艺术不怎么

感兴趣 (1) (2) (3) (4) (5)

6)... 开朗，社交能力强 (1) (2) (3) (4) (5)

7)... 喜欢寻找别人

的缺点 (1) (2) (3) (4) (5)

8)... 工作细致周到 (1) (2) (3) (4) (5)

9)... 容易紧张或焦虑 (1) (2) (3) (4) (5)

10)... 想象力丰富 (1) (2) (3) (4) (5)

*Optional extra agreeableness item:-*

11)... 是一个替人考虑为人和善的人

For the English-language BFI-10, see Rammstedt and John [10].
